# Activation of GLP-1R modulates the spontaneous discharge of nigral dopaminergic neurons and motor behavior in mice with chronic MPTP Parkinson's disease

**DOI:** 10.3389/fnagi.2025.1529919

**Published:** 2025-04-25

**Authors:** Wen-Hong Liu, Cui Liu, Yan Xue, Xiang-Rong Sun, Xin-Yi Chen, Lei Chen

**Affiliations:** ^1^Department of Physiology and Pathophysiology, School of Basic Medicine, Qingdao University, Qingdao, China; ^2^Department of Histology and Embryology, School of Clinical and Basic Medical Sciences, Shandong First Medical University and Shandong Academy of Medical, Jinan, China; ^3^Department of Neurology, Affiliated Hospital of Qingdao University, Qingdao, China

**Keywords:** GLP-1, firing activity, dopaminergic neuron, MPTP, Parkinson's disease

## Abstract

The gradual decline of nigral dopaminergic neurons is the main cause of Parkinson's disease (PD), yet as of now, there exists no conclusive therapeutic intervention. Glucagon-like peptide-1 (GLP-1) is an incretin, which is also a key substance regulating neuronal activity and synaptic transmission. GLP-1 receptors (GLP-1Rs) are widely expressed in the central nervous system. Chronic administration of low doses of 1-methyl-4-phenyl, 1,2,3,6-tetrahydropiridine (MPTP) mitigates mortality in mice during the modeling phase, thereby more closely mirroring the progression of PD. This study aims to observe the effects of GLP-1 receptor agonists (GLP-1RAs) on the firing activity of nigral dopaminergic neurons and motor behaviors in MPTP-induced chronic PD mice. Our findings revealed that peripheral administration of GLP-1RAs exendin-4 significantly alleviated motor impairments in MPTP-induced chronic PD mice. Concurrently, peripheral administration of exendin-4 increased the number of active dopaminergic neurons, improved the spontaneous firing activity, as well as alleviated MPTP-induced dopaminergic neuron loss in MPTP-induced PD mice. Furthermore, local administration of exendin-4 directly increased the firing rate of nigral dopaminergic neurons via GLP-1Rs, suggesting that peripheral administration of exendin-4 may exert neuroprotection through its mild excitation on dopaminergic neurons. These findings collectively imply that peripheral administration of GLP-1RAs may hold potential in the treatment of PD.

## 1 Introduction

Parkinson's disease (PD) is the second most common neurodegenerative disorder next to Alzheimer's disease (AD), affecting 3% of the population aged over 65 and up to 5% of those over 85, which is increasingly becoming a global concern (Hill et al., 2023; Reddy et al., 2024; Wang et al., 2023). The typical symptoms of PD include motor disturbances (tremors), rigidity, bradykinesia and difficulty in walking, while non-motor symptoms encompass autonomic dysfunction (such as urinary incontinence and orthostatic hypotension), emotional and cognitive impairments (such as depression and memory loss), as well as sleep disorder. Although the degeneration of dopaminergic neurons is the primary hallmark of PD, other hallmarks (such as α-Syn accumulation in Lewy bodies, neuroinflammation) are also particularly important for the appearance of symptoms (Liu et al., 2022b; Nechushtai et al., 2023). Dopamine in the midbrain is primarily synthesized by dopaminergic neurons in the substantia nigra and the ventral tegmental area. The progressive degeneration of these dopaminergic neurons leads to a reduction in dopamine levels within the substantia nigra and striatum, triggering the onset of motor symptoms associated with PD (Zhou et al., 2023).

The spontaneous firing activity of nigral dopaminergic neurons alters under parkinsonian state. The abnormal neuronal firing activity includes decreased firing frequency, reduced number of active neurons, increased burst firing and increased firing frequency (Chen et al., 2023b). In a genetic mitochondrial model of PD, three types of dopaminergic neurons with different firing activity, similar firing rate to control, no spontaneously firing, increased firing rate, were identified (Good et al., 2011). It is reported that mild excitatory stimuli can enhance the survival of dopaminergic neurons in the substantia nigra as well as the expression of tyrosine hydroxylase (TH) (Salthun-Lassalle et al., 2004). Optogenetic excitation of dopaminergic neurons in fruit flies can significantly delay the degenerative changes in dopaminergic neurons induced by electrical knockout (EKO, which activates potassium channels in dopaminergic neurons, thereby reducing their excitability) and improve motor deficits (Qi et al., 2017). It can be seen that increasing the excitability of dopaminergic neurons in the substantia nigra within a certain range can protect the neurons and improve the motor impairments in animal models of PD.

Glucagon-like peptide-1 (GLP-1) is a hormone primarily secreted by the enteroendocrine cells of the distal ileum and colon. It stimulates glucose-dependent insulin secretion and typically plays a role in blood glucose regulation and the reduction of hepatic fat (Newsome and Ambery, 2023). GLP-1 is also expressed in the central nervous system (CNS), primarily released by the spontaneously firing proglucagon (PPG) neurons in the nucleus of the solitary tract (NTS; Perez-Leighton et al., 2024). GLP-1 receptors (GLP-1Rs) are widely expressed in the brain, serving as a key substance in the regulation of neuronal activity and synaptic transmission (Neha et al., 2024). When bound to GLP-1Rs, GLP-1 can exert various biological effects through multiple signaling pathway including thermogenesis, regulation of feeding, neurogenesis, blood pressure regulation, mood modulation and energy homeostasis (Ghosh et al., 2023; Farkas et al., 2021; Katsurada and Yada, 2016). Given that GLP-1 has a short half-life, typically ranging from 1 to 2 min, many GLP-1 receptor agonists (GLP-1RAs) have been developed to provide the functions of GLP-1 (Abushamat et al., 2024). Exenatide is a short-acting GLP-1RAs that was first introduced by Astra Zeneca in 2005 and is synthesized from exendin-4, with a half-life of approximately 2.4 h (Ma et al., 2021; Alavi et al., 2019). Both exendin-4 and exendin (9–39) can bind to GLP-1Rs and are commonly used in research as agonists and antagonists of GLP-1Rs (Göke et al., 1993).

Clinical studies have shown that GLP-1RAs improve cerebral metabolism in patients with AD and delay the progression of motor symptoms in PD (Athauda et al., 2017; Gejl et al., 2016). Moreover, electrophysiological studies have confirmed that activation of GLP-1Rs can increase neuronal electrical activity through both presynaptic and postsynaptic mechanisms, thereby regulating various functions in the CNS. For example, administering exogenous GLP-1RAs to the ventral tegmental area or activating endogenously released GLP-1 using optogenetic techniques can inhibit the intake of high-fat food (Wang et al., 2015). In addition, activation of GLP-1Rs can increase the neuronal spontaneous firing rate of nucleus accumbens postsynaptically, thereby inhibiting cocaine-seeking behavior in rats (Hernandez et al., 2019). Morphological studies have revealed that GLP-1Rs are expressed in the substantia nigra (Farkas et al., 2021). However, long-term GLP-1 intervention on motor behaviors and spontaneous firing activity of nigral dopaminergic neurons in PD remain unclear. In this study, we aim to observe the effects of exogenous GLP-1 on the spontaneous firing activity of nigral dopaminergic neurons by using *in vivo* extracellular single-unit electrophysiological techniques, as well as its regulation on motor behaviors.

## 2 Materials and methods

### 2.1 Animals

Adult male C57BL/6 mice (8–10 weeks, 22–26 g) were purchased from Ji'nan Pengyue Laboratory Animal Breeding CO., Ltd., and housed on a 12/12 h light/dark cycle (light-dark, 07:00 light on, 19:00 light off) with food and water *ad libitum*. The animal experiments were approved by the Animal Ethics Committee of Qingdao University (approval No.202303C5780202406109) on September, 2022. During the experiments, all efforts were taken to minimize animal discomfort.

### 2.2 Experimental design

The MPTP-induced chronic PD mouse model was established in male mice. These mice were randomly assigned to one of three groups: control group (CON, *n* = 12), MPTP-induced chronic PD group (MPTP, *n* = 68), and exendin-4 treatment group (MPTP+EX-4, *n* = 22). Exendin-4 (Tocris, UK) was dissolved in vehicle to make the stock solution (100 μg/mL exendin-4). MPTP (Sigma, USA) was injected intraperitoneally at a dose of 25 mg/kg for 5 consecutive weeks (3 days per week for MPTP; to ensure that the mice received the same level of stimulation, vehicle was given on the remaining 4 days of the week) to induce PD mice (Meredith et al., 2008), and control group mice were given an equal amount of vehicle at the same time every day. In the exendin-4 treatment group, mice received intraperitoneal MPTP 3 days a week for 5 consecutive weeks, while also receiving intraperitoneal exendin-4 (50 μg/kg/day) daily (Zhang et al., 2021). Before preparing MPTP model, the mice were trained in open-field and rotarod apparatus. The mice were acclimated to the animal housing facility for 7 days before the experiment began. Behavioral tests started the day after completing of administration. A total of 16 mice were used for behavioral experiments (5 in CON group, 6 in MPTP group and 5 in MPTP+EX-4 group). A total of 78 mice were used in the present electrophysiological experiments (8 in CON group, 56 in MPTP group and 14 in MPTP+EX-4 group).

### 2.3 Immunohistochemistry

The process for TH immunohistochemical staining was same as previously described (Liu et al., 2018b). Briefly, 25-μm-thick brain sections were incubated overnight at 4°C with primary antibody against TH (1:800, GeneTex, China) and then with HRP conjugated goat anti-rabbit IgG (PV6001, Zhongshan Golden Bridge, China) at room temperature. Afterwards, the sections were rinsed, dehydrated and cover slipped. According to the anatomical atlas the substantia nigra pars compacta (SNc) was identified. Every fourth section from the region between bregma −2.92 mm to −3.28 mm was analyzed. Four sections per brain were stained and quantified. The counting frame was 50 × 50 μm. Cell counts were performed at 200 × magnification. The number of TH-ir neurons was the sum of all the 4 sections counted.

### 2.4 Open field test

The open field test can detect the spontaneous motor activity and exploratory behaviors of mice (Peng et al., 2019). Mice were placed in the center of a square arena (40 cm long × 40 cm wide × 35 cm high), and allowed to move freely during 10-min recording. Seventy percentage alcohol was used to clean the field after each test. The total traveling distance and the percentage of time in central zone of chamber were monitored and analyzed (Zhang et al., 2018).

### 2.5 Rotarod test

The rotarod test was used to evaluate the motor coordination of the mice. The black sensor plate was placed horizontally at the bottom of the rotating rod device and the mice were placed in individual channels on the rotating rod. On clicking START, the rotation bar began to rotate, and the speed gradually increased from 4 to 40 r/min. The time at which each mouse fell from the rotating rod was recorded. The rod automatically stopped rotating at 300 s. For mice that did not fall, the time recorded was 300 s (Deacon, 2013).

### 2.6 Single unit *in vivo* extracellular electrophysiological recordings

Mice were anesthetized with 20% urethane (1 g/kg) intraperitoneally and placed in a stereotaxic apparatus (Narishige SN-3, Tokyo, Japan). The degree of anesthesia was sufficient to eliminate pinch withdrawal. According to the stereotaxic atlas, a craniotomy was performed (AP: 2.9–3.6 mm, ML: 1.0–1.5 mm, and DV: 4.0–4.5 mm). Three-barrel microelectrodes were pulled via a multipipette puller (51,517, Stoelting, USA). One of the three barrels served as the recording electrode. The other two were filled with the following drugs: (i) exendin-4 (10 μM) and vehicle; (ii) exendin (9–39; 10 μM) and vehicle; (iii) exendin-4 and exendin (9–39), which were connected to a cell microinjector (LPP01-100, LongerPump, USA). The recording location and the electrophysiological features of neurons were used to distinguish the dopaminergic neurons from GABAergic neurons (Liu et al., 2022a). The electrodes were carefully positioned within the SNc. As the SNc is adjacent to the substantia nigra pars reticulata, usually only one dopaminergic neuron was recorded in each track. The specific electrophysiological features are more important to distinguish nigral dopaminergic neurons from nigral GABAergic neurons. The dopaminergic neurons display an obvious biphasic discharge with a long duration and a notch during the rising phase, accompanied by a low frequency (< 8 Hz) (Grace and Bunney, 1984). The nigral GABAergic neurons exhibit a higher firing rate (>10 Hz) than nigral dopaminergic neurons and a shorter discharge duration without a notch (Wilson et al., 1977). The electrical signals of neurons were amplified by an amplifier (1,700, A-M system, USA) and passed through a low (5,000 Hz) and high (1 Hz) pass filters. The spike data were analyzed via Spike 2 software (Cambridge Electronic Design Limited, Cambridge, UK). After stable recording for at least 5 min, drugs were ejected onto the surface of the neurons. The basal firing rate was determined by the average frequency of 120 s baseline data before drug administration. The maximal change of firing rate within 50 s following drug application was considered as drug effect. The threshold of significance of firing rate was established based on deviations from a mean firing rate. The changes in firing rate exceeded 2 standard deviations (SDs) of basal firing rate were considered significant. The ratio of the SD of inter spike interval (ISI) to its mean was identified as the coefficient of variation (CV), an indicator of the regularity of firing activity. After the electrophysiological recordings, mice were transcardially perfused with normal saline and 4% paraformaldehyde solution, and then the brains were sectioned into 50-μm-thick coronal sections to observe the position of the electrodes.

### 2.7 Drugs and statistics

Exendin-4 (1,933/1) was obtained from Tocris (UK). Exendin (9–39; HY-P0264) was obtained from MedChemExpress (USA). MPTP was purchased from Sigma (USA). The sample size for electrophysiological recording was calculated by pilot studies according to the formula for paired samples, while behavioral tests were determined based on that of previous study (Liu et al., 2018a). The data were expressed as mean ± SEM. The comparison of the firing rate before and after drug application was carried out by Paired *t*-test. Before the *t*-test, the Kolmogorov-Smirnov test was performed to determine if the data were normally distributed. The comparison of means among multiple groups was performed using One-Way Analysis of Variance (One-Way ANOVA). Mann-Whitney test was used to compare the difference of the change in firing rate between two groups. The level of significance was preset using a *p*-value of 0.05.

## 3 Results

### 3.1 Peripheral administration of GLP-1ras ameliorated motor impairments in MPTP-induced chronic PD mice

**Figure 1:**
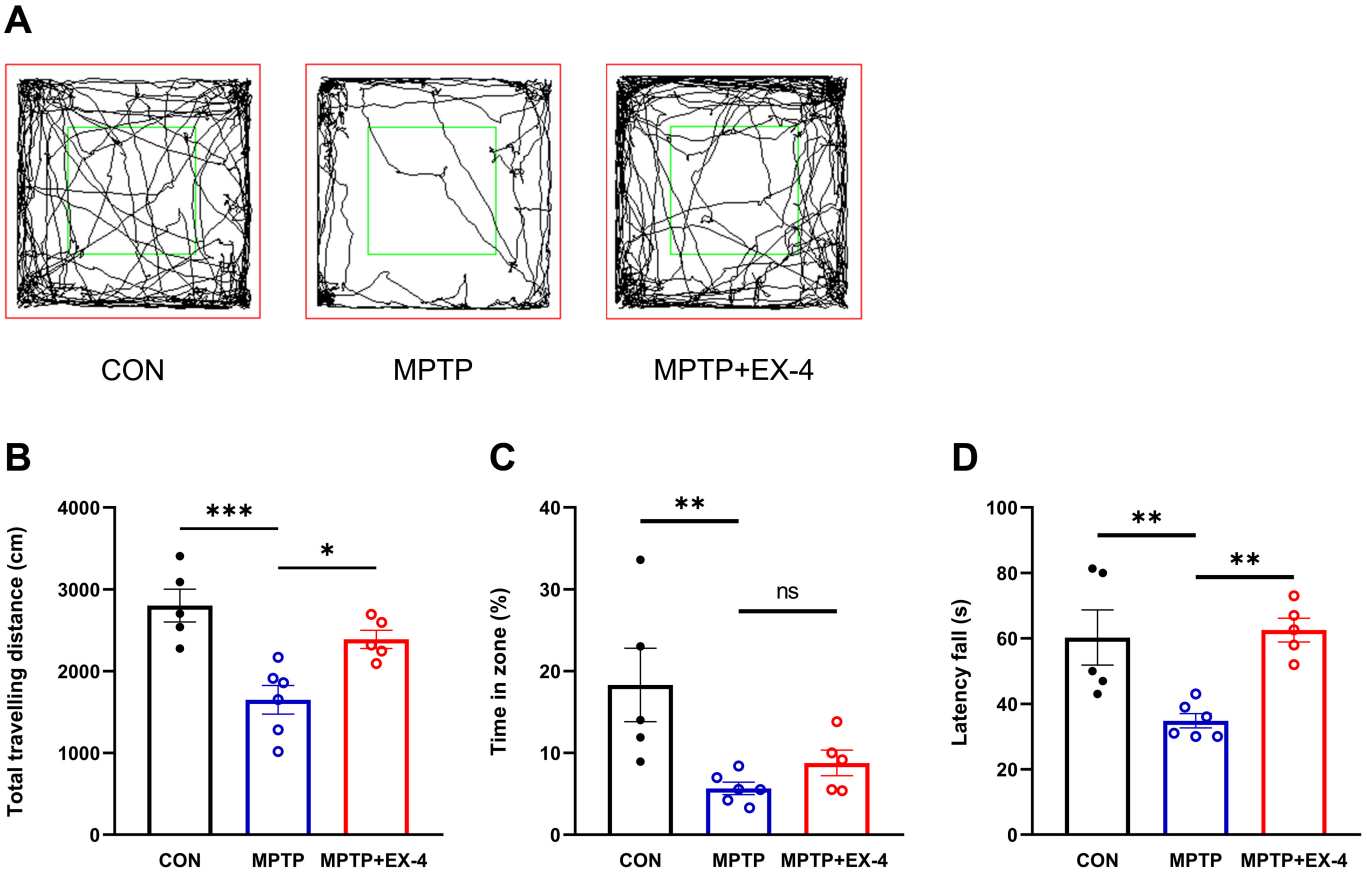
Effects of intraperitoneal administration of exendin-4 on motor impairments in chronic MPTP mice. **(A)** Schematic diagrams of the motor traces of three group mice in the open field test. **(B)** Pooled data showing the effects of peripheral administration of exendin-4 on the total traveling distance in open field test. **(C)** Pooled data showing the effects of peripheral administration of exendin-4 on the time in central zone in open field test. **(D)** Pooled data showing the effects of peripheral administration of exendin-4 on the latency time in rotarod test in MPTP-induced chronic PD mice. *P < 0.05, **P < 0.01, ***P < 0.001, one-way ANOVA followed by LSD *post-hoc* test. ns, not significant.

GLP-1 exerts a variety of biological effects *in vivo* by binding to GLP-1Rs. Due to the short half-life of naturally occurring GLP-1 in the human body, which makes it susceptible to degradation, we selected the GLP-1RAs exendin-4, characterized by a longer half-life and the ability to cross blood-brain barrier (BBB), to investigate the effects of long-term peripheral GLP-1RAs exendin-4 intervention on motor behaviors in MPTP-induced chronic PD mice. Mice in MPTP group demonstrated significantly reduced total traveling distance (CON group: 2,802.16 ± 200.26 cm, MPTP group: 1,648.86 ± 174.84 cm, *P* = 0.0006, Figures 1A, B), and percentage of time in central zone (CON group: 18.30 ± 4.49%, MPTP group: 5.66 ± 0.75%, *P* = 0.0078, Figures 1A, C) compared to CON group in the open field test. In contrast, compared to MPTP group, mice in MPTP+EX-4 group exhibited improved performance in the open field test (total traveling distance: 2,389.44 ± 111.54 cm, *P* = 0.0145, Figures 1A, B), although there was no significant difference in the percentage of time in central zone (8.77 ± 1.57%; *P* = 0.6172, Figures 1A, C). Furthermore, similar results were observed in the rotarod test (Figure 1D), where the MPTP group mice remained on the rod for 34.83 ± 2.21 s, significantly less than that of CON group (60.27 ± 8.40 s, *P* = 0.0067). Notably, mice in the MPTP+EX-4 group showed significant improvement (62.53 ± 3.61 s, *P* = 0.0037) compared to MPTP group.

### 3.2 Peripheral administration of GLP-1ras modulated the firing activities of nigral dopaminergic neurons in MPTP-induced chronic PD mice

**Figure 2:**
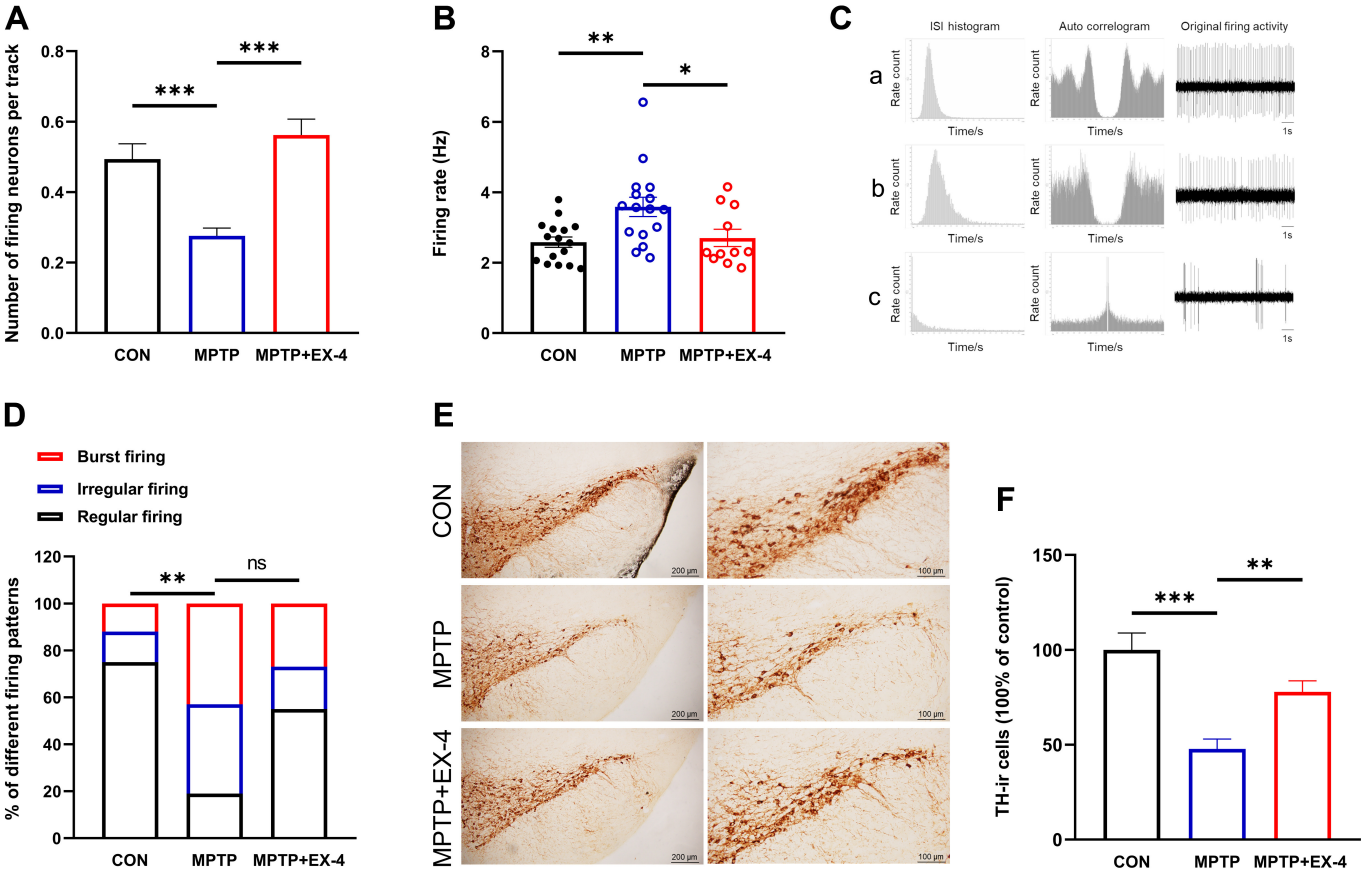
Effects of intraperitoneal administration of exendin-4 on the spontaneous discharge of nigral dopaminergic neurons and TH expression in chronic MPTP mice. **(A)** Effects of long-term peripheral exendin-4 intervention on the quantity of spontaneously active nigral dopaminergic neurons. **(B)** The impact of long-term peripheral exendin-4 intervention on the baseline firing rate of nigral dopaminergic neurons. **(C)** Three firing patterns of nigral dopaminergic neurons in mice. The upper (a), middle (b) and lower (c) traces show typical regular, irregular and burst firing neurons, respectively. The regular pattern is characterized by a symmetrical distribution of ISI and at least three identifiable peaks in the auto-correlogram. The irregular firing pattern is characterized by Poisson's distribution of ISI and < 2 peaks in the auto-correlogram. The burst firing pattern is characterized by the positive skew distribution of ISI and an auto-correlogram with no peaks or a single initial peak. **(D)** The influence of long-term peripheral exendin-4 intervention on the firing patterns of nigral dopaminergic neurons. **(E)** Effects of intraperitoneal administration of exendin-4 on the expression of TH in the substantia nigra in chronic MPTP mice. Photomicrographs on the right corresponded to the left pictures. **(F)** Quantitative analysis of TH-ir neurons in the SNc. *P < 0.05, **P < 0.01, ***P < 0.001, one-way ANOVA with the Bonferroni *post-hoc* test. ns, not significant.

In the preceding section, peripheral GLP-1RAs exendin-4 intervention can improve motor deficits in MPTP-induced chronic PD mice. To further investigate the underlying mechanisms of GLP-1, we subsequently examined the effects of peripheral exendin-4 intervention on the electrophysiological activity of nigral dopaminergic neurons in MPTP-induced chronic PD mice. The spontaneous firing activities of nigral dopaminergic neurons from the CON, MPTP, and MPTP+EX-4 groups were recorded by using *in vivo* extracellular electrophysiological techniques. Initially, a significant reduction in the number of spontaneously active dopaminergic neurons was observed in MPTP group. Specifically, mice in CON group had an average of 0.49 neurons recorded per track, whereas the MPTP group exhibited an average of 0.28 neurons recorded per track (*t* = −0.2179, *P* = 0.0005, Figure 2A). In contrast, the MPTP+EX-4 group demonstrated an average of 0.56 neurons recorded per track (*t* = −0.2861, *P* < 0.0001, compared to the MPTP group, Figure 2A). Simultaneously, the spontaneous firing rate of nigral dopaminergic neurons in MPTP group (3.59 ± 0.27 Hz) was significantly increased compared to that in CON group (2.58 ± 0.15 Hz; *t* = −3.221, *P* = 0.0041, Figure 2B), while the mean firing rate in MPTP+EX-4 group (2.70 ± 0.24 Hz) was decreased comparted to that in MPTP group (*t* = −2.272, *P* = 0.0239, Figure 2B).

When observing the spontaneous discharge activity of dopaminergic neurons in the SNc, three different discharge patterns were recorded. According to the CV of the ISI and the autocorrelation diagram of the discharge interval, they can be divided into regular discharge, irregular discharge and bursting discharge (Figure 2C). The firing patterns of spontaneously active dopaminergic neurons in the MPTP group exhibited significant alterations compared to the CON group (*P* = 0.006, Figure 2D). These alterations were characterized by an increase in the proportion of burst and irregular firing patterns, along with a decrease in the proportion of regular firing patterns. Notably, long-term peripheral exendin-4 intervention induced a shift of firing patterns to more regular although there was no significant difference (*P* = 0.149, Figure 2D). Further immunohistochemical staining showed that the number of TH-ir neurons in MPTP group was 47.83 ± 5.16% of that in CON group (*P* < 0.0001, compared to CON group, Figures 2E, F), application of exendin-4 increased the number of TH-ir neurons to 77.89 ± 5.82% of that in control group (*P* = 0.0096, compared to MPTP group, Figures 2E, F).

### 3.3 Nigral application of GLP-1ras increased the spontaneous firing rate of dopaminergic neurons in MPTP-induced chronic PD mice

**Figure 3:**
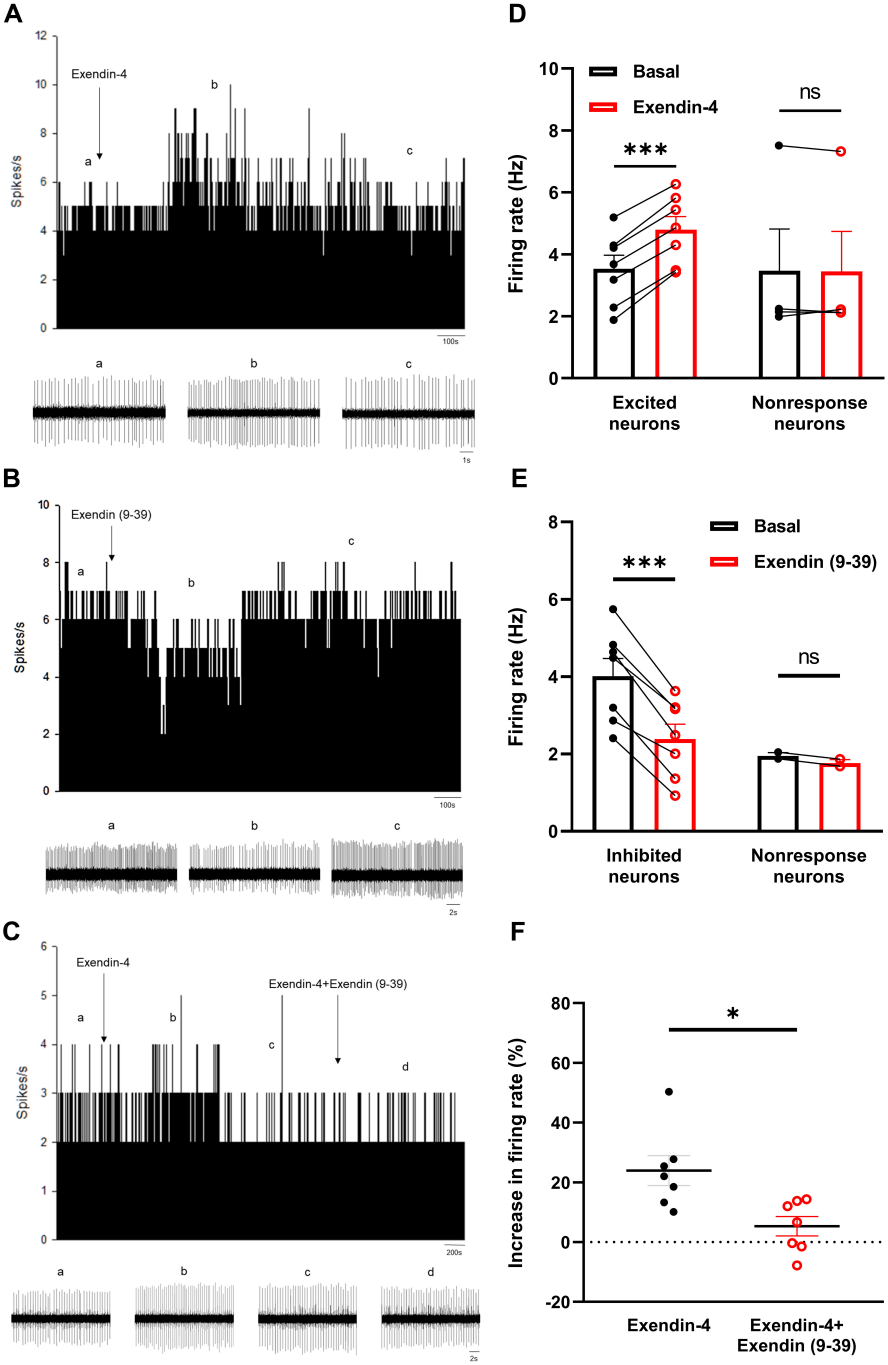
Electrophysiological effects of exendin-4 administered to nigral dopaminergic neurons in chronic MPTP mice. Typical rate histogram showing the effects of exendin-4 **(A)**, exendin (9–39) **(B)** and co-application of exendin-4 and exendin (9–39) **(C)** on the spontaneous firing rate of a nigral dopaminergic neuron in MPTP-induced chronic PD mice. The bottom traces show the original spikes at different phases (control, drug effects and recovery) of the experiment. **(D)** Summarized data showing that exendin-4 increased the firing rate of nigral dopaminergic neurons. **(E)** Pooled data showing that exendin (9–39) decreased the firing rate of nigral dopaminergic neurons. **(F)** Pooled data showing the increase in firing rate between exendin-4 alone and co-application of exendin (9–39) and exendin-4. *P < 0.05, ***P < 0.001, paired *t*-test and Mann-Whitney test. ns, not significant.

Previous studies have observed the expression of GLP-1Rs in a subset of nigral dopaminergic neurons of normal mice. Accordingly, we investigated the effects of administering exendin-4 to the neuronal surface on the spontaneous firing activity of dopaminergic neurons in MPTP-induced chronic PD mice, with the results presented in Figure 3. Exendin-4 was administered via micro pressure injection using *in vivo* electrophysiological techniques. Notably, the firing rate of 7 out of the 11 recorded neurons exhibited a significant increase from 3.53 ± 0.44 Hz to 4.80 ± 0.42 Hz (*t* = 17.61, *P* < 0.0001, Figures 3A, D). The firing rate of the remaining 4 neurons showed no significant change, with the variation in firing rate induced by exendin-4 was < 2 SDs above the mean of the baseline firing rate (baseline: 3.47 ± 1.35 Hz, exendin-4: 3.46 ± 1.29 Hz, Figure 3D).

Considering that PPG neurons in the NTS and the olfactory bulb can synthesize and secrete GLP-1 and GLP-1 secreted by intestinal L cells can cross BBB to enter the CNS, we subsequently examined whether endogenous GLP-1 participates in the increase of spontaneous firing activity of nigral dopaminergic neurons in MPTP-induced chronic PD mice. The specific GLP-1R antagonist exendin (9–39) was applied to the surface of dopaminergic neurons. In MPTP group, exendin (9–39) decreased the firing rate in 7 out of the total 9 neurons from 4.02 ± 0.46 Hz to 2.39 ± 0.38 Hz (*t* = 9.279, *P* < 0.0001, Figures 3B, E). The firing rate of the remaining 2 neurons showed no significant change [baseline: 1.96 ± 0.08 Hz, exendin (9–39): 1.77 ± 0.09 Hz, Figure 3E].

**Figure 4:**
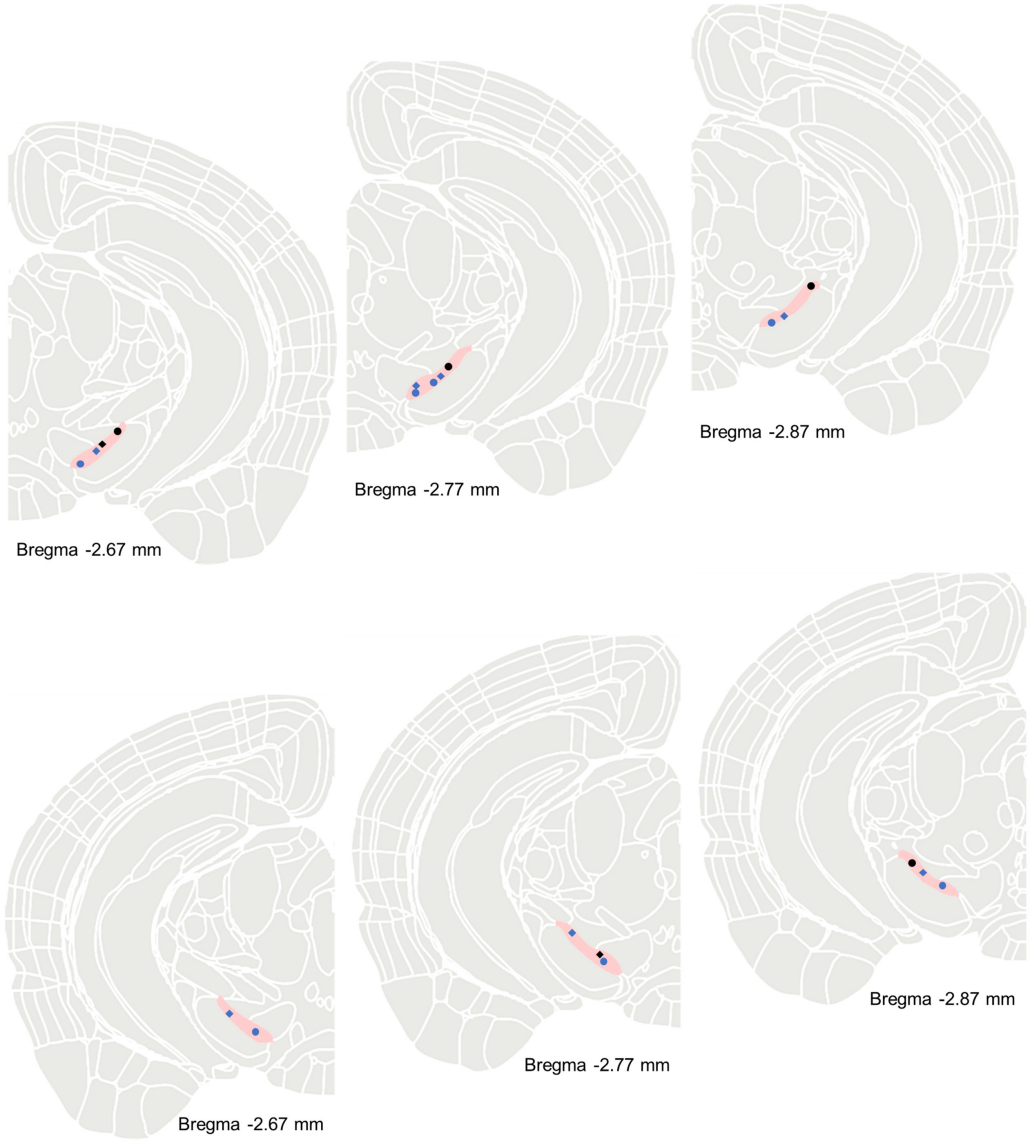
Maps of recorded neurons in MPTP-induced chronic PD mice excited by exendin-4 (blue circle, *n* = 7) or non-response to exendin-4 (black circle, *n* = 4), and inhibited by exendin (9–39) (blue rhombus, *n* = 7) or non-response to exendin (9–39; black rhombus, *n* = 2) in SNc.

Subsequently, we investigated the receptor mechanisms underlying the excitatory effects of the GLP-1RAs exendin-4 on nigral dopaminergic neurons in MPTP-induced chronic PD mice. During the experimental procedure, after recording spontaneous firing activity from the identified dopaminergic neurons, we initially administered exendin-4 via micro pressure injection to ascertain its effect on modulating the firing rate of the neurons. Once the excitatory effects induced by exendin-4 returned to baseline levels, we then co-administered exendin-4 and the exendin (9–39) to determine whether exendin (9–39) would block the excitatory effects of exendin-4 on neuronal electrical activity (Figure 3C). The results indicated that exendin (9–39) effectively blocked the increase in the firing rate of nigral dopaminergic neurons induced by exendin-4. Among the 7 recorded neurons, administration of exendin-4 alone resulted in an 23.91 ± 5.00% increase in firing rate (Figure 3F). Concurrent administration of exendin-4 and exendin (9–39) only induced 5.30 ± 3.27% change in firing rate (Z = −2.492, *P* = 0.011). These findings suggest that the excitatory effects of exendin-4 on nigral dopaminergic neurons of MPTP-induced chronic PD mice are mediated through GLP-1R activation. Figure 4 showed the maps of recorded neurons with or without response to exendin-4 or exendin (9–39) in SNc.

## 4 Discussion

### 4.1 Peripheral GLP-1ras intervention alleviated motor deficits in MPTP-induced chronic PD mice

In the early 1980s, researchers discovered GLP-1 while investigating diabetes and blood glucose regulation (Holst, 2007). Through years of rigorous exploration, scientists established that GLP-1 can reduce blood glucose levels in humans, prompting pharmaceutical companies to investigate its potential as a treatment for diabetes. Currently, GLP-1 analogs are widely used in clinical practice for the management of type 2 diabetes and weight control (Nauck et al., 2021). Furthermore, a study examining whether GLP-1 analogs could slow the progression of kidney disease in diabetic patients was prematurely halted due to positive outcomes (Hernandez et al., 2018; Mann et al., 2017; Gerstein et al., 2019). Clinical trials are also underway assessing the efficacy of GLP-1 medications in the treatment of AD and PD. Initial findings suggest that GLP-1RAs may delay the progression of motor symptoms in patients with PD (Athauda et al., 2017; Gejl et al., 2016). Given that GLP-1 medications are typically administered via peripheral injection in clinical settings, and acknowledging the capability of the GLP-1RAs exendin-4 to traverse the BBB, this study employed peripheral GLP-1 interventions to observe their effects on PD mice. Furthermore, as the prolonged systemic administration of low-dose MPTP does not result in increased mortality rates in young adult or aged mice, and more closely resembles the gradual progression characteristic of neurodegenerative processes, this therapeutic approach can be employed to simulate various stages of PD. This model facilitates a better understanding of the disease's pathophysiology and promotes the testing of protective and restorative therapies (Munoz-Manchado et al., 2016). Consequently, in this study, we utilized MPTP-induced chronic PD mice for our experiments. We conducted behavioral assessments following long-term peripheral administration of the GLP-1RAs exendin-4 to MPTP-induced chronic PD mice. The results demonstrated that peripheral GLP-1 intervention significantly increased the total traveling distance of mice in the open field test, as well as extended the time spent by the mice on the rotating rod during the rotarod test. These findings suggest that peripheral GLP-1 administration can ameliorate the motor impairment symptoms exhibited by MPTP-induced chronic PD mice.

### 4.2 Peripheral GLP-1ras intervention improved the discharge activities of nigral dopaminergic neurons in MPTP-induced chronic PD mice

Early studies have identified that specific deficits in the electrophysiological characteristics of neurons may lead to various neurodegenerative diseases (Klassen et al., 2011; Tai et al., 2014). In the context of PD, the excitability of nigral dopaminergic neurons begins to decline gradually prior to neuronal death and the onset of motor impairment symptoms (Hirsch et al., 1988). Previous study revealed three types of nigral dopaminergic neurons with different firing frequency, similar firing rate to control, no spontaneously firing and increased firing rate, in a model of Parkinson's disease. The majority of neurons are silent neurons which could be driven to fire in a normal pacemaker firing fashion by adequate stimulation, suggesting that these neurons might be in the early phase of disease (Good et al., 2011). Another report noted that optogenetic excitation of dopaminergic neurons in fruit flies could ameliorate motor deficits (Qi et al., 2017), suggesting that GLP-1 may improve motor symptoms in MPTP-induced PD mice by increasing the electrical activity of nigral dopaminergic neurons. Consequently, we employed single unit *in vivo* electrophysiological techniques to record the spontaneous firing activity of nigral dopaminergic neurons following peripheral exendin-4 intervention in MPTP-induced chronic PD mice. Chronic application with exendin-4 significantly increased the number of active nigral dopaminergic neurons suggesting that exendin-4 may drive the large number of silent dopaminergic neurons to fire and therefore could protect the neurons in not yet irreversible condition. Consistently, the present TH immunostaining results also showed that exendin-4 reduced the loss of dopaminergic neurons.

In present study as well as our previous study (Bian et al., 2021), the basal firing rate of dopaminergic neurons in MPTP mice is higher than that of normal mice. We hypothesized that as the large number of dopaminergic neurons change to silent under parkinsonian state, the recorded neurons are mainly from the reported normal firing rate neurons and overactive neurons (Good et al., 2011), and therefore with increased mean firing frequency. Treatment with exendin-4 may drive the silent neurons to fire in a normal firing fashion and therefore the recorded average firing rate coming from the three types of firing neurons is reduced than that of MPTP alone group. Moreover, although no statistically significant differences were observed when comparing the neuronal firing patterns between MPTP group and exendin-4 treatment group, exendin-4 induced an observable shift of firing patterns to more regular in MPTP-induced chronic PD mice. These findings suggest that peripheral GLP-1RAs may activate PPG neurons within the NTS via vagal afferent fibers, subsequently projecting either directly or indirectly to brain regions that express GLP-1Rs. This mechanism may contribute to the reduction of neuronal tonic firing, thereby delaying degenerative changes.

### 4.3 Nigral GLP-1ras increased the spontaneous firing rate of nigral dopaminergic neurons in MPTP-induced chronic PD mice

Recent studies have indicated that GLP-1RAs may exert neuroprotective effects in neurodegenerative diseases such as PD (Glotfelty et al., 2020; Chen et al., 2023a; Nowell et al., 2023). For instance, GLP-1RAs have been shown to increase dopamine levels in the striatum and alleviate motor deficits in animal models of PD (Dahiya et al., 2022). Previous studies demonstrated that mild excitatory stimuli can enhance the survival of dopaminergic neurons in the substantia nigra as well as the expression of TH (Michel et al., 1999). To provide direct evidence that the present peripheral administration of exendin-4 exerts neuroprotection through its mild excitation on the dopaminergic neurons, we next locally administering exendin-4 to see the direct effects on the firing activity of dopaminergic neuron. Electrophysiological recordings demonstrated that application of exendin-4 to the surface of neurons significantly increased the firing rate of a subset of nigral dopaminergic neurons. Previous studies have reported that neurons exhibiting excitatory effects by exendin-4 are preferentially located in the medial aspect of the SNc. Furthermore, in the context of PD, exendin-4 continues to exert a similar excitatory effect on nigral dopaminergic neurons, which aligns with our experimental findings. Given that the impaired spontaneous firing activity of dopaminergic neurons may contribute to the pathogenesis of PD, we hypothesize that the neuroprotective properties of exendin-4 on these neurons may be related to its excitatory effects. Given that endogenous GLP-1 is primarily synthesized and secreted by PPG neurons in the NTS, we also investigated the effects of endogenous GLP-1 on the spontaneous firing activity of nigral dopaminergic neurons in MPTP-induced chronic PD mice. Our experiments revealed that application of the specific GLP-1R antagonist exendin (9–39) to the surface of nigral dopaminergic neurons significantly reduced the firing rate of a subset of these neurons. This finding suggests that endogenous GLP-1 plays an excitatory role in the spontaneous firing activity of nigral dopaminergic neurons.

This study first observed that peripheral GLP-1RAs exendin-4 intervention improved motor deficits in MPTP-induced chronic PD mice, reduced the loss of spontaneously active nigral dopaminergic neurons, and enhanced their firing activity. The current results suggest the existence of a subset of nigral dopaminergic neurons in MPTP-induced chronic PD mice that exhibit suppressed excitability. Upon delivering excitatory stimuli to these neurons, it is possible to restore them to a normal physiological state, thus improving the spontaneous firing activity of dopaminergic neurons in the PD mice. This presents a potential therapeutic target for mitigating or halting the progression of PD. The novelty of this experiment lies in the use of exendin-4 administered peripherally to assess its effects on motor behavior and the discharge activity of dopaminergic neurons in MPTP-induced chronic PD mice. The results indicate that peripheral exendin-4 intervention may enhance the excitability of nigral dopaminergic neurons, representing a potential therapeutic approach for PD. However, this study has limitations, as we did not evaluate the long-term effects of the peripheral GLP-1R antagonist exendin (9–39) on motor behavior and the firing activity of dopaminergic neurons in MPTP-induced chronic PD mice, nor did it compare the excitatory effects of peripheral exendin-4 on normal mice vs. MPTP-induced chronic PD mice.

## 5 Conclusion

In conclusion, the present study demonstrates that long-term peripheral administration of the GLP-1RAs exendin-4 alleviated motor deficits and improved the firing activities of nigral dopaminergic neurons in MPTP-induced chronic PD mice. Furthermore, direct activation of nigral GLP-1Rs enhances the spontaneous firing activity of nigral dopaminergic neurons in MPTP-induced chronic PD mice, while blockade of endogenous GLP-1Rs led to the inhibition of the spontaneous discharge activity of nigral dopaminergic neurons. These findings indicate that peripheral GLP-1 intervention may enhance the excitability of nigral dopaminergic neurons, representing a potential therapeutic strategy for the treatment of PD.

## Data Availability

The original contributions presented in the study are included in the article/supplementary material, further inquiries can be directed to the corresponding authors.
